# An evidence-based guide to the efficacy and safety of isometric resistance training in hypertension and clinical implications

**DOI:** 10.1186/s40885-022-00232-3

**Published:** 2023-03-15

**Authors:** Biggie Baffour-Awuah, Melissa J. Pearson, Gudrun Dieberg, Jonathan D. Wiles, Neil A. Smart

**Affiliations:** 1 Clinical Exercise Physiology, School of Science and Technology, University of New England, Armidale, Australia; 2 Sports Sciences, School of Psychology and Life Sciences, Canterbury Christ Church University, Canterbury, UK

**Keywords:** Exercise, Isometric resistance training, Blood pressure, Hypertension

## Abstract

More than 30 randomized controlled trials, supported by individual patient-level and group-level meta-analyses and a Delphi analysis of expert opinion, unequivocally show isometric resistance training (IRT) elicits antihypertensive benefits in healthy people and those with chronic illness. We aim to provide efficacy and safety evidence, and a guide for IRT prescription and delivery. Recommendations are made for the use of IRT in specific patient populations and appropriate methods for IRT delivery. Published data suggest IRT consistently elicits mean blood pressure reductions of 7.4/3.3 mmHg systolic blood pressure/diastolic blood pressure, equivalent to antihypertensive medication monotherapy. Blood pressure reductions of this size are associated with an approximate 13% to 22% reduction in major cardiovascular events. Moreover, IRT is safe in a range of patient populations. We suggest that IRT has the greatest potential benefit when used as an antihypertensive therapy in individuals unwilling and/or unable to complete aerobic exercise, or who have had limited adherence or success with it; individuals with resistant or uncontrolled hypertension, already taking at least two pharmacological antihypertensive agents; and healthy or clinical populations, as an adjunct to aerobic exercise and dietary intervention in those who have not yet attained control of their hypertension. IRT is efficacious and produces clinically meaningful blood pressure reductions (systolic blood pressure, 7 mmHg; diastolic blood pressure, 3 mmHg). IRT is safe and typical program delivery requires only about 17 min weekly. IRT should be used as an adjunct to other exercise modalities, in people unable to complete other types of exercise, or in resistant hypertension.

## Background

In the last two decades more than 30 randomized controlled trials (RCTs) [1–16], supported by individual patient-level [17] and group-level meta-analyses [18–26], have unequivocally demonstrated that isometric resistance training (IRT) elicits antihypertensive benefits in people who are healthy [8, 9, 14, 15] and those who exhibit prehypertension [2, 10], hypertension [1, 11, 13, 16], peripheral artery disease [4], coronary heart disease [3] and heart failure [5]. Current guidelines suggest the gold standard exercise prescription for managing hypertension is aerobic exercise [27] with emphasis more recently attributed to IRT [28, 29]. While recognizing current guidelines, Cornelissen and Smart [30] first demonstrated, using a pooled data analysis, that reductions in systolic blood pressure (SBP), diastolic blood pressure (DBP), and mean arterial pressure (MAP), following a 4 to 8 weeks program of IRT, are greater than those observed with an aerobic exercise training (AET) or dynamic resistance training (DRT) program of similar duration [30].

IRT has also been shown to elicit improvements in endothelial function [4, 31–34] and coronary collateral vessel blood flow in people with coronary artery disease [35]. IRT can achieve these beneficial physiological adaptations while eliciting a much lower rate pressure product (RPP) index than both AET and DRT [36]. RPP is the product of SBP and heart rate (HR), and cardiologists often use this as an estimate of myocardial oxygen uptake (clinically, RPP is a reliable indicator of myocardial oxygen demand and valuable marker of cardiac function). In cardiac patients diagnosed with exertional symptoms, such as angina, a RPP threshold can be set to avoid symptom exacerbation and related adverse events, such as exercise-induced arrhythmia. During isometric contractions there are only small increases in HR, and pressor responses are often smaller than when performing recommended levels of AET and DRT, resulting in a lower RPP with IRT [36]. Intuitively, this would mean that IRT is less likely to provoke exertional stress and cardiac events than AET or DRT. Early IRT cohort studies [37–40] in the 1970s used very high exertional intensity and evoked hypertensive responses [37] that suggested IRT may be dangerous [37, 41], especially for those with hypertension. IRT studies in the last 30 years have used exertional intensities mostly in the range of 30% of an individual’s maximum capacity and this has led to well documented antihypertensive effects [17] and abrogation of safety concerns related to IRT [19].

As the use of IRT to treat hypertension, is a relatively new concept, there remain, understandably, concerns amongst exercise specialists and other health professionals about whether this form of therapy can be used safely and in which patient populations. Even though more than 30 RCTs since the 1990s have suggested a sustained post training antihypertensive benefit [42], and the initial scientific opinion, to avoid IRT, has reversed somewhat in the last two decades, this form of therapy remains under-utilized and some bodies still have reservations about its use [43].

The aim of this work is to provide a systematic summary and practical guide to IRT, based upon the available published evidence for efficacy and safety.

### Isometric resistance training

**Fig. 1:**
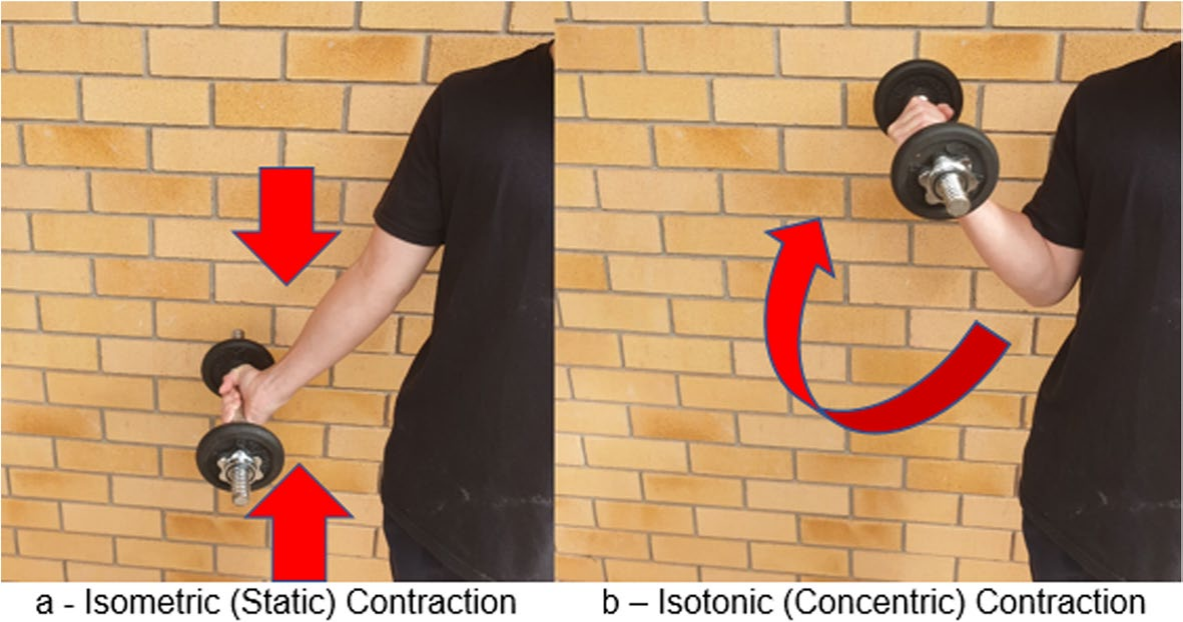
Visual depiction of (**A**) isometric and (**B**) isotonic muscular contractions (arrows indicate arm movement; no movement (**A**) and flexion (**B**)). During an isometric contraction, muscle length does not change; however, tension might increase. During an isotonic contraction, muscle length either shortens (concentric contraction) or lengthens (eccentric contraction) and tension remains the same

An isometric muscle contraction is one where there is no change in muscle length, but a force is applied, and muscle tension may increase (Fig. 1A). This may occur because the force produced by the muscle(s) is the same as the external resistance (e.g., holding a dumbbell) or because the opposing load is greater than the force generated (e.g., trying to push over a large, stable tree trunk). As there is no observable change in joint angle and muscle length during the contraction, it is often referred to as a “static” contraction. Simply, dynamic or isotonic contractions (concentric and eccentric) move loads, while isometric contractions create force and/or tension without movement.

During isometric work the contraction is static and sustained and therefore the intramuscular pressure generated is often enough to partially or fully inhibit vascular blood flow in the vessels that serve that particular muscle or muscle group [36]. For example, handgrip squeezing exercise requires a static forearm contraction and therefore the flow in the brachial artery may be partially or fully occluded. The extent of the disruption to blood flow is dependent on the relative intensity of the contraction [39, 44]. After the isometric contraction ceases reactive hyperemia occurs; this is transient blood flow, at a raised rate, that occurs in healthy blood vessels following an ischemic episode, such as IRT at a sufficient proportion or percentage of maximal voluntary contraction (MVC; the maximal force-generating capacity of a muscle or group of muscles in humans), usually this is 15% to 30% of MVC [14, 45]. This reactive hyperemia is facilitated by vasodilation, and this results in a sustained blood pressure reduction that lasts for an undefined period of time but commonly several hours [46]. As with other types of exercise training, repeated exposure to cycles of ischemia-hyperemia-reperfusion can lead to stable blood pressure changes (lowering) which are sustained if one continues to train. This training status dependent lowering of blood pressure has been shown to be a result of anatomical increases in blood vessel diameter with aerobic exercise [47], but similar data is not available for IRT. However, IRT causes an acute stimulation of the metaboreflex in an attempt to restore muscle blood flow. This and other long-term physiological responses (occurring from approximately 4 weeks onwards) may reduce tissue oxidative stress, and improve vascular endothelial function, baroreflex sensitivity, and autonomic balance [48].

To put this into context, a healthy man aged 20 to 50 years would be expected to generate a maximal isometric handgrip contraction of about 40 to 50 N. It is thought that at about 20% to 30% of one’s handgrip MVC, there is a measurable disruption to blood flow. For lower limb isometric contractions (e.g., squatting with one’s back resting against a wall with knees at a 90° angle), it is believed that the disruption to blood flow occurs at a slightly lower relative intensity of 15% to 25% MVC; this is probably due to the greater force generated and specific fiber arrangement [49] in the muscles of the lower limb. The relative exercise intensity (equivalent to %MVC) generated in isometric wall squats can be adjusted by changing the knee joint angle [50, 51].

A rapidly growing body of evidence continues to highlight the benefits of IRT in lowering blood pressure (BP). However, to date, the exact mechanisms responsible for the BP lowering effects of IRT have not been fully elucidated. The effects of IRT most likely involve interactions of a number of pathways and mechanisms, including endothelial function, structural vascular adaptations, oxidative stress, and autonomic nervous system activity [13, 52].

## Efficacy of IRT

**Fig. 2:**
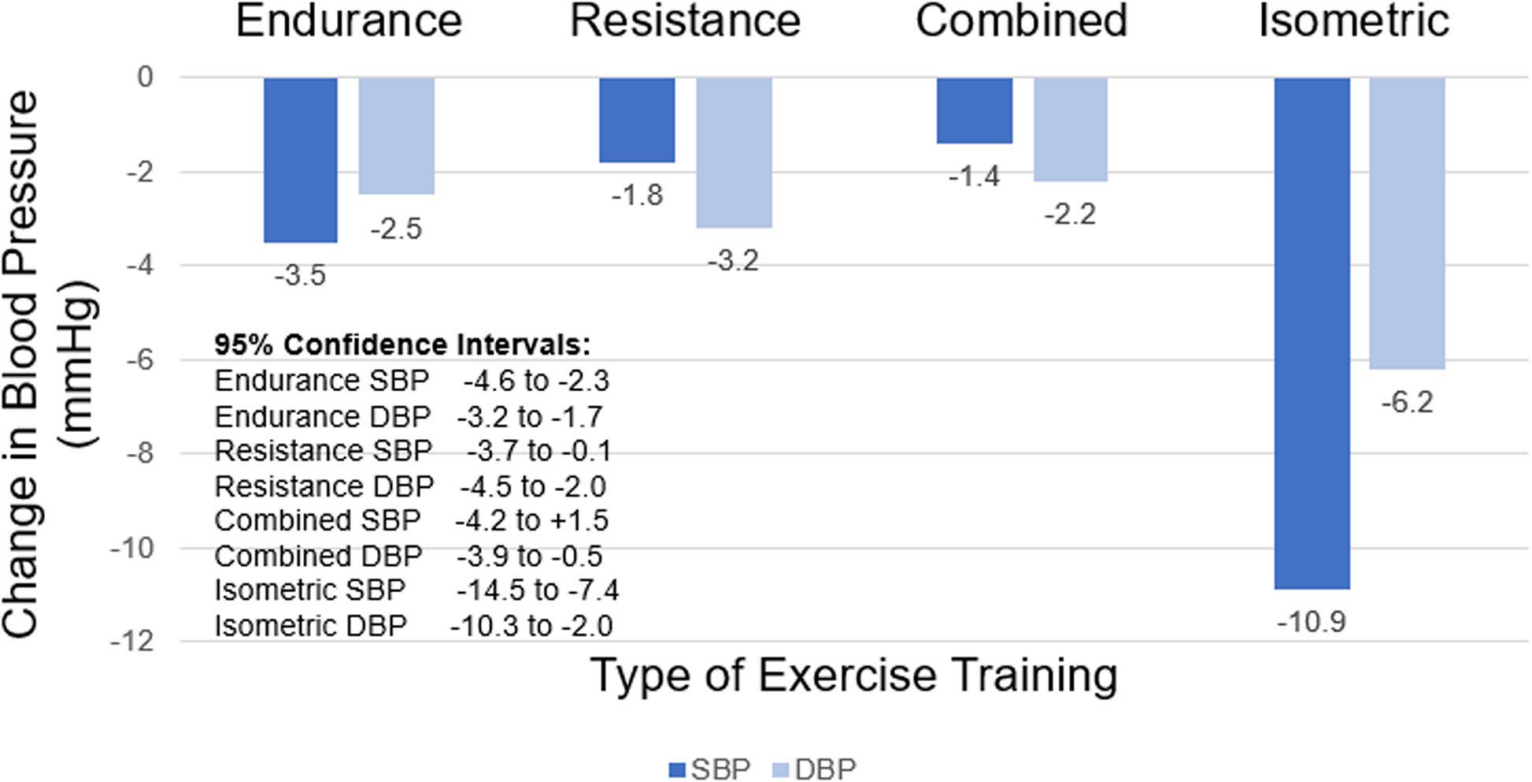
Change in systolic blood pressure (SBP) and diastolic blood pressure (DBP) by exercise modality. Based on Cornelissen and Smart [30]

A 2013, meta-analysis showed typical changes in BP for published studies of exercise training with a training duration of 8 to 12 weeks (Fig. 2, based on Cornelissen and Smart 2013) [30]. The sizes of these differences are both statistically significant and clinically meaningful. There was no overlap in 95% confidence intervals (CIs) between IRT and the other three types of exercise training; however, there was considerable overlap in the 95% CIs for the three other types of exercise (Fig. 2).

In 2014, a group-level meta-analysis was conducted on all nine published RCTs of IRT (223 participants) and confirmed that the antihypertensive effects were greater than those reported in most aerobic and DRT studies [18]. In progressing the evidence base, a 2016 group-level meta-analysis was performed of all 11 published RCTs of IRT. Again, the analyses confirmed antihypertensive effects, the additional findings from this work were that certain patient subgroups may benefit more [20]. Specifically, people over 45 years appear to benefit more than younger people, and people diagnosed with hypertension appeared to derive a greater hypertensive benefit than those who were normotensive [20]. This work also suggested a greater benefit in IRT trials lasting > 8 weeks, compared to shorter studies.

In 2019 an individual patient data (IPD) meta-analysis was performed on 12 randomized, controlled trials of IRT [17]. The purpose of taking the IPD approach was that, while retaining the characteristics of individual study designs (e.g., exercise program variables), it was possible to make direct comparisons between subject characteristics and effect sizes. The IPD methodology is considered superior to group-level meta-analyses [53–55]. This IPD analyses confirmed similar antihypertensive effect sizes for SBP (–7.35 mmHg; 95% CI, –8.95 to –5.75 mmHg) and DBP (–3.29 mmHg; 95% CI, –5.12 to –1.46 mmHg), with very small 95% CIs, that were observed in previous systematic reviews and pooled analyses in the period of 2013 to 2016. The size of this effect is thought to be similar to the antihypertensive benefit elicited from taking one antihypertensive medication [56].

Most recently, in 2021 a Delphi method was employed to seek expert consensus-building on items related to the safety, efficacy, and delivery of IRT, in light of the current trend towards use of IRT as an adjunct treatment for hypertension [57]. This study showed experts’ consensus that IRT is as efficacious as an antihypertensive therapy. Furthermore, the antihypertensive efficacy of IRT has stood the test of time over the last three decades.

## Safety

### Cardiovascular responses to exercise

An intensity-dependent rise in HR and BP occurs with all types of physical exercise. Central command, muscle reflexes, and the arterial baroreflex are important regulators of the cardiovascular system during exercise [58, 59]. An exaggerated BP response to exercise is a predictor of future chronic hypertension which may lead to risk of organ damage, cardiac events, and mortality [60–62]. Somewhat paradoxically regular exercise, at an appropriate intensity and volume, has been shown to reduce BP and reduce the risk of organ damage and cardiovascular disease. As with many novel medical treatment approaches there is often an inherent concern that new ideas may cause harm. In hypertensive individuals compared to normotensive individuals, it is this “possible” exaggerated pressor (hypertensive) response, resulting in an increase in pressure load on the heart, that appears to raise the most concern among professionals in relation to the safety of IRT. However, as outlined below, evidence indicates that the acute increase in BP during IRT is no larger, and in fact in some studies smaller [36, 63, 64], than that observed with AET.

We acknowledge that the threshold for an extreme or exaggerated hypertensive response does vary with different datasets [65]. The threshold for a hypertensive response to physical stress is as low as 190/90 mmHg and as high as 250/120 mmHg in different settings [65]. The physiological basis of why isometric contractions at or above 50% MVC may cause extreme hypertensive responses was outlined in the early 1970s. In 1970, Lind [41] described that at 10% to 15% MVC, isometric contractions can be maintained for 30 min or more. At these low tensions, the HR increases by only a few beats per minute (bpm) and BP by only 5 to 15 mmHg, for as long as the tension is maintained. Muscle blood flow also increases to a steady state, which corresponds with the indefinite period contractions can be held for. At 20% MVC and higher, fatigue occurs rapidly. At 20% MVC, contractions can be held for around 10 min. At 30% MVC, the duration is about 5 min, while at 50% MVC, it is 1 to 2 min. When working to a constant force above 20% MVC, the blood flow in the muscle does not reach a steady state, nor does the HR or BP. Instead, all these factors continue to increase until fatigue, as a function of duration intervenes. At the point of fatigue, it is uncommon for HR to be over 120 bpm. However, both SBP and DBP increase dramatically and almost in parallel, and MAP at fatigue is commonly 140 mmHg or more. By contrast, in fatiguing rhythmic exercise (a treadmill test of progressive severity), the HR characteristically reaches near maximum values, while the MAP shows only slight changes. In isometric exercise, widespread peripheral vasoconstriction occurs, so increased cardiac output then elicits a BP rise, but stroke volume does not increase and at higher tensions (for example, 50% MVC) it decreases, so that increased HR is the sole contributor to the increase in cardiac output [41].

For the reasons outlined in the previous paragraph, there was a historical reluctance to use IRT in people with even mild hypertension, but recent trials have used low to moderate training intensities (10%–30% MVC) [2, 9] normally performed for a short 2-min duration; these are associated with, relatively modest [64] rises in BP during IRT. In summary, during both isotonic and isometric muscular contractions there is a notable pressor (BP raising) response at intensities of 50% MVC or above, but this is mitigated by the lower (10%–30% MVC) training intensities and shorter durations that have become common practice. Finally, one should not forget that there is also evidence of blood flow impairment with repeated concentric contractions [66].

### Early concerns about IRT safety

As mentioned previously, from a historical perspective IRT was contraindicated for people with hypertension [67, 68]. In some of the early studies, IRT was employed at a relatively high proportion (> 50%) of MVC, with some studies asking research subjects to contract at maximal capacity (100% MVC) resulting in an exaggerated pressor response [69, 70]. Contractions 50% MVC, or above, are likely to generate a notable pressor (hypertensive) response > 200/100 mmHg and should be avoided in all but young, healthy people clinically free of cardiovascular disease.

**Fig. 3:**
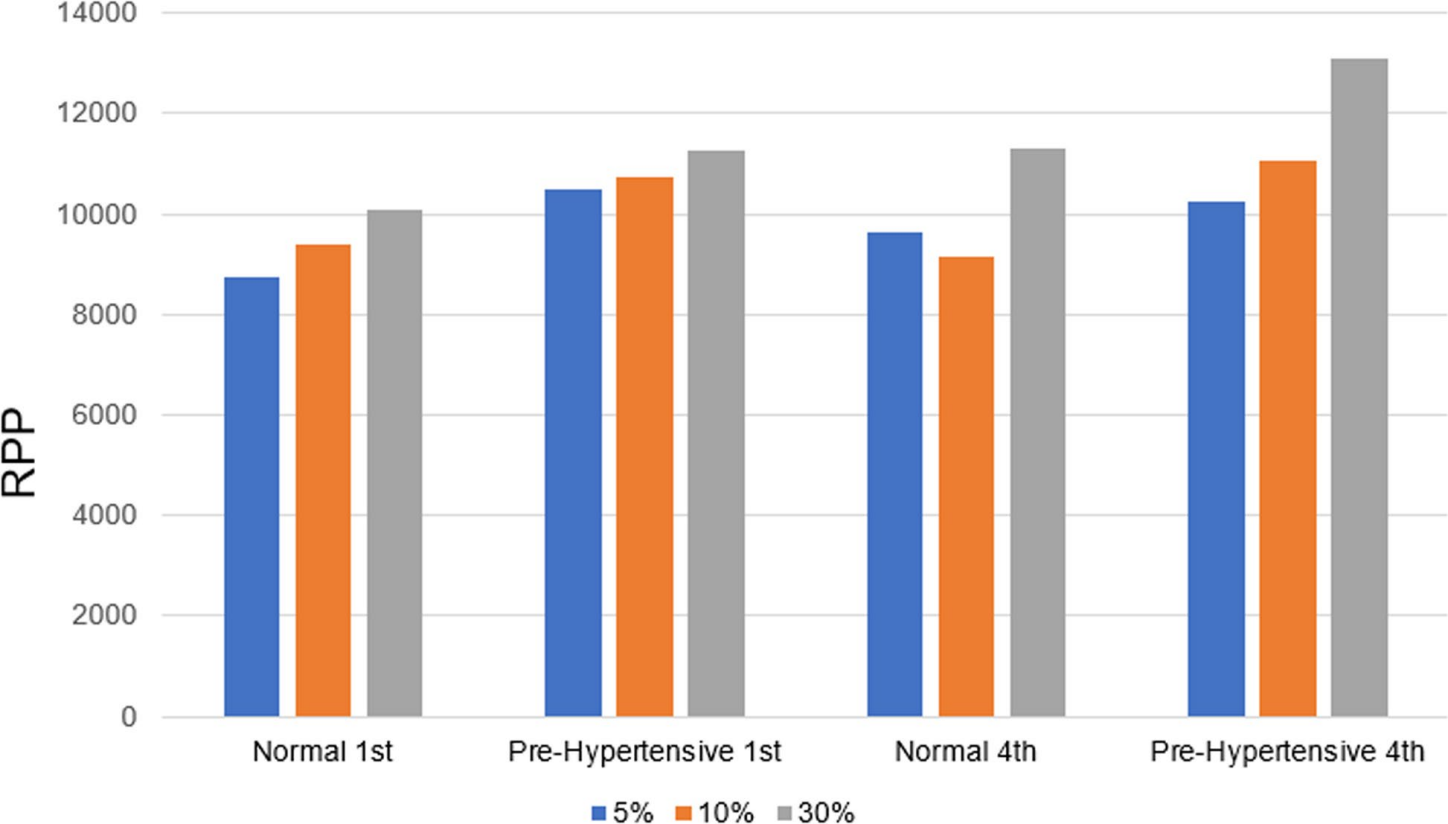
Peak rate pressure product across isometric handgrip exercise at various maximal voluntary contraction [36]. Rate pressure product (RPP) values following the first and fourth isometric resistance training bout in normotensive and prehypertensive populations at 5%, 10% and 30% maximal voluntary contraction (MVC)

One should point out that once IRT ceases, BP will return very close to baseline levels in a matter of seconds [71, 72], whereas the normal time course of recovery to prolonged aerobic exercise is of longer duration, usually several minutes, but can be several hours [73]. Previous work has identified that people are at a raised risk of adverse exertional-related health events during aerobic exercise and the 30 to 60 min post exercise recovery period [74]. Three key points are evident from our observations of BP responses to IRT. First, one can see from Fig. 3 that RPP exhibits a positive linear correlation with %MVC during IRT activity. Second, despite a slight cumulative upward drift, the RPP values still remain less that the RPP values for moderate aerobic intensity in hypertensive individuals at equivalent %MVC (Fig. 3, Table 1). Third, individuals with hypertension exhibit slightly higher RPP values than people with normal BP, at the same %MVC intensity (Table 1).

**Table 1:** Comparative RPP responses in IRT versus aerobic exercise [36]

RPP response	Normotensive	Hypertensive
Aerobic (moderate intensity)		
Heart rate rises (bpm)	40–60	40–60
Blood pressure rises (mmHg)	40–60	50–80
RPP	140 × 180 = 25,200	140 × 200 = 28,000
IRT (30% MVC)		
Heart rate rises (bpm)	5–10	5–10
Blood pressure rises (mmHg)	20–30	40–50
RPP	80 × 130 = 10,400	80 × 140 = 11,200
RPP difference	14,800	16,800

RPP is calculated as heart rate × systolic blood pressure

*RPP* Rate pressure product, *bpm* beats per minute, *IRT* Isometric resistance training, *MVC* Maximal voluntary contraction

### Current evidence for IRT safety

A recently published meta-analysis [19] of RCTs of IRT showed long-term BP lowering effects are achieved with isometric contractions at 15% to 30% MVC. While contractions at this intensity do raise BP, the increase is much smaller (20–30 mmHg) than would be seen with moderate intensity aerobic exercise. IRT also results in much smaller (10–30 bpm) increases in HR. The net RPP is much lower with IRT versus aerobic exercise (Table 1). Available data suggests that this difference is greatest when participants use small muscle mass isometric handgrip training, compared to a RPP value of 18,000 bpm × mmHg (based upon BP and HR average over the last 30 s of the final/fourth wall squat bout) for participants (with high-normal BP) performing double-leg IRT [64]. RPP is used primarily by cardiologists to estimate oxygen utilization in the myocardial tissues. Often people with symptoms of angina pectoris become symptomatic at a reproducible threshold [75]. In people with exertional angina symptoms, or cardiac arrhythmia, an RPP threshold can be set in order to minimize symptom exacerbation and lower the risk of serious cardiovascular events.

IRT produces an ischemic preconditioning response that is cardio protective [3, 76]. This effect is also seen with aerobic and possibly dynamic resistance exercise, but a much higher RPP is generated during these latter two forms of exercise. IRT produces a reactive hyperemic response immediately post exercise that improves endothelial function long-term [73]. IRT elicits greater uptake of blood into the coronary collateral circulation in people with coronary artery disease [35]. Similarly, in people with peripheral arterial disease, IRT was reported to reduce brachial DBP, and increase flow mediated dilatation [4], a measure of endothelial function. IRT also elicits rises in vascular endothelial growth factor (stimulant for new blood vessel growth) in heart failure patients [5]. The available evidence therefore suggests that the “default” position that IRT is unsafe is perhaps unsupported, and indeed contradicted somewhat, by the published literature. Benefits can also be seen in other patient populations, as shown in Table 2, which provides a summary of analyses and studies across different populations indicating any safety concerns or possible contraindications to the use of IRT.

**Table 2:** Evidence for efficacy and safety in various patient populations in which published trial data exists for acute and chronic BP responses to IRT

Analyses/trial & condition	Evidence	BP reductions SBP/DBP (mmHg) & other findings	Contra-indicated
**Meta-analyses**			
Meta-analyses -Hypertension -Prehypertension -Normotensive	9 Meta-analyses: Carlson et al. 2014 [18] Hansford et al. 2021 [19] Inder et al. 2016 [20] Jin et al. 2017 [21] Kelly et al. 2010 [22] Kelly et al. 2021 [23] Loaiza-Betancur et al. 2020 [24] Lopez-Valenciano et al. 2019 [25] Owen et al. 2010 [26] 1 IPD meta-analysis: Smart et al. 2019 [17]	Long-term anti-hypertensive effect Average reduction: –7 / –4 –6.22 / –2.78	No adverse events related to IRT reported from any included trials
**Recent RCTs & conditions**			
Prehypertension	*N* = 400 (Ogbutor et al. 2019) [10]	–7.48 / –6.41	No adverse events related to IRT
Hypertensive (non-medicated and medicated)	6 Trials *N* = 33 (Cahu Rodrigues et al. 2020) [1] *N* = 40 (Punia et al. 2020) [12] *N* = 22 (Okamoto et al. 2019) [11] *N* = 24 (Taylor et al. 2019) [13] *N* = 44 (Yoon et al. 2019) [16] *N* = 40 (Ahmed et al. 2019) [7]	–16 / –8 –8.75 / –8.35 –17 / –7 –12.3 / –6.2 –8.9 / –5.6 –18.75 / –15.5 & improved arterial stiffness	No adverse events related to IRT reported in any study
Peripheral artery disease	*N* = 102 (Correia et al. 2020) [4]	–6 / –3 & reduced flow mediated dilatation	No adverse events related to IRT
Coronary heart disease	*N* = 55 (Chen et al. 2019) [3]	–10.32 / –5.63 & improved VEGF levels	No adverse events related to IRT
Phase II/III cardiac rehabilitation patients	*N* = 11 (Gordon et al. 2019) [6] (18% had heart failure)	–16 / –9 in 50% of participants & improved VEGF levels	No adverse events related to IRT; IRT may not be effective immediately following (12 wk) a cardiac event
Heart failure	*N* = 30 (Gao et al. 2018) [5]	Improved VEGF levels	No adverse events related to IRT
Healthy	*N* = 20 (Herrod et al. 2019) [8]	–7.2 / –6	No adverse events related to IRT

For the purposes of this work “long-term BP” means sustained (not post exercise hypotension) changes induced following an IRT program of 3 weeks or more that were measured > 12 h after the last IRT bout

*BP* Blood pressure, *IRT* Isometric resistance training, *SBP* Systolic blood pressure, *DBP* Diastolic blood pressure, *IPD* Individual patient data, *RCT* Randomized clinical trial, *VEGF* Vascular endothelial growth factor

While the benefits of IRT can be seen in a range of patient populations, concerns about safety of this type of exercise may be further alleviated if one recalls IRT is only sustained for 1 to 2 min, which is below the 3-min threshold scientists believe is necessary to develop reperfusion injury [77]. With 2 to 3 min rest periods for IRT the minimal increase in HR and mild/moderate hypertensive responses are brief. IRT protocols allow for adequate (complete) recovery periods, which are achieved in seconds with IRT and not minutes as with aerobic exercise. We have reported the findings from IRT training and studies below. A detailed summary of analyses and recent RCTs can be found in Table 2.

## Clinical implications

### Clinical relevance of IRT

Current evidence indicates IRT lowers BP, but one may ask if these changes are clinically meaningful and hence supportive of the transition of IRT into “real world” clinical practice. In order to provide a “clinical” meaning, we conducted secondary analyses from our earlier IPD and categorized participants as responders versus nonresponders [17]. We defined “responders” as any participant who showed a 5 mmHg or greater reduction in SBP, a 3 mmHg or greater reduction in DBP, and/or a 3 mmHg or greater reduction in MAP [78].

For each of SBP, DBP, and MAP, we calculated responder rate, absolute risk reduction (ARR) and number needed to treat (NNT). The 28.1% ARR is the difference between the proportion of responders in the IRT (55.5%) minus control (27.4%). The NNT is the inverse of the ARR. Approximately three times as many people who undertake IRT experience a favorable BP lowering response compared to controls who do not undertake IRT. This means that about one-quarter (27.4%) of people will lower their BP independent of IRT, probably due to any of the following approaches, or combination thereof—medication changes, weight loss, or improved diet. Moreover, if someone with hypertension undertakes a program of IRT then their likelihood of reducing their BP increases to about 55% (Table 3).

**Table 3:** IRT responder rates, absolute risk reduction, and number needed to treat [17]

	Responder rate (*n* = 191)	Absolute risk reduction (%)	NNT (95% CI)
Systolic blood pressure	106 (55.5)	28.1	4 (2.80–7.42)
Diastolic blood pressure	92 (48.2)	20.1	5 (3.22–11.10)
Mean arterial pressure	105 (55.0)	30.4	4 (2.80–7.42)

Values are presented as number (%) or number only

*NNT* Number needed to treat, *CI* Confidence interval

A landmark meta-analysis by Xie et al. [79] demonstrated that if one were able to reduce a participant’s SBP by 7 mmHg this would equate to a 13% risk reduction for myocardial infarction and a 22% risk reduction for stroke. Moreover, our 2019 IPD meta-analysis [17] demonstrates that this magnitude of BP reduction is possible to detect in a sample of > 320 participants, as demonstrated in our two-step model of our IPD data. Additionally, the recent Delphi study of experts’ consensus fully supports IRT as it produces clinically meaningful BP reductions [57].

### Transition of IRT into clinical practice

#### Who is most likely to benefit from IRT?

IRT has been safely conducted with no adverse events reported to date (Table 2). The only contraindication to IRT identified is that there may be a raised risk of mild cognitive impairment in postmenopausal women who have a history of preeclampsia [80]. It should be clarified though, that this possible association was not generated from a longitudinal follow-up study design that allowed direct causation to be attributed.

As a guide to exercise practitioners who may wish to use IRT in their patients, the recommendations listed below may help to optimize IRT utilization. While IRT can be used in most people, it is suggested that IRT has the greatest potential benefit when utilized as an antihypertensive therapy in: (1) individuals unwilling or unable to complete aerobic exercise, or who have had limited adherence or success with it; (2) individuals with resistant or uncontrolled hypertension, already taking at least two pharmacological antihypertensive agents; (3) healthy or clinical populations, as an adjunct to aerobic exercise and dietary intervention in those who have yet to attain control of their hypertension.

#### What are the other potential applications for IRT use?

Recent work has postulated that IRT may also be beneficial for managing BP in patients with mild cognitive impairment as vascular integrity is likely improved. There is the potential for IRT to initiate a cascade of vascular, neurotrophic, and neuroendocrine events that could enhance cognitive function [81].

In healthy pregnant women, testing of maternal and fetal hemodynamic responses to IRT has indicated no specific safety concerns, concluding that isometric exercise may facilitate exercise compliance in pregnant women [82]. Given the benefits of IRT in hypertensive individuals, future work examining IRT in pregnant women who exhibit prehypertension during pregnancy, may indicate beneficial effects on BP, and this may possibly prevent progression to hypertension and the incidence of preeclampsia in later stages in some individuals. However, future work is required in this area before using IRT in this population.

#### How should the exercise specialist triage people to IRT?

**Fig. 4:**
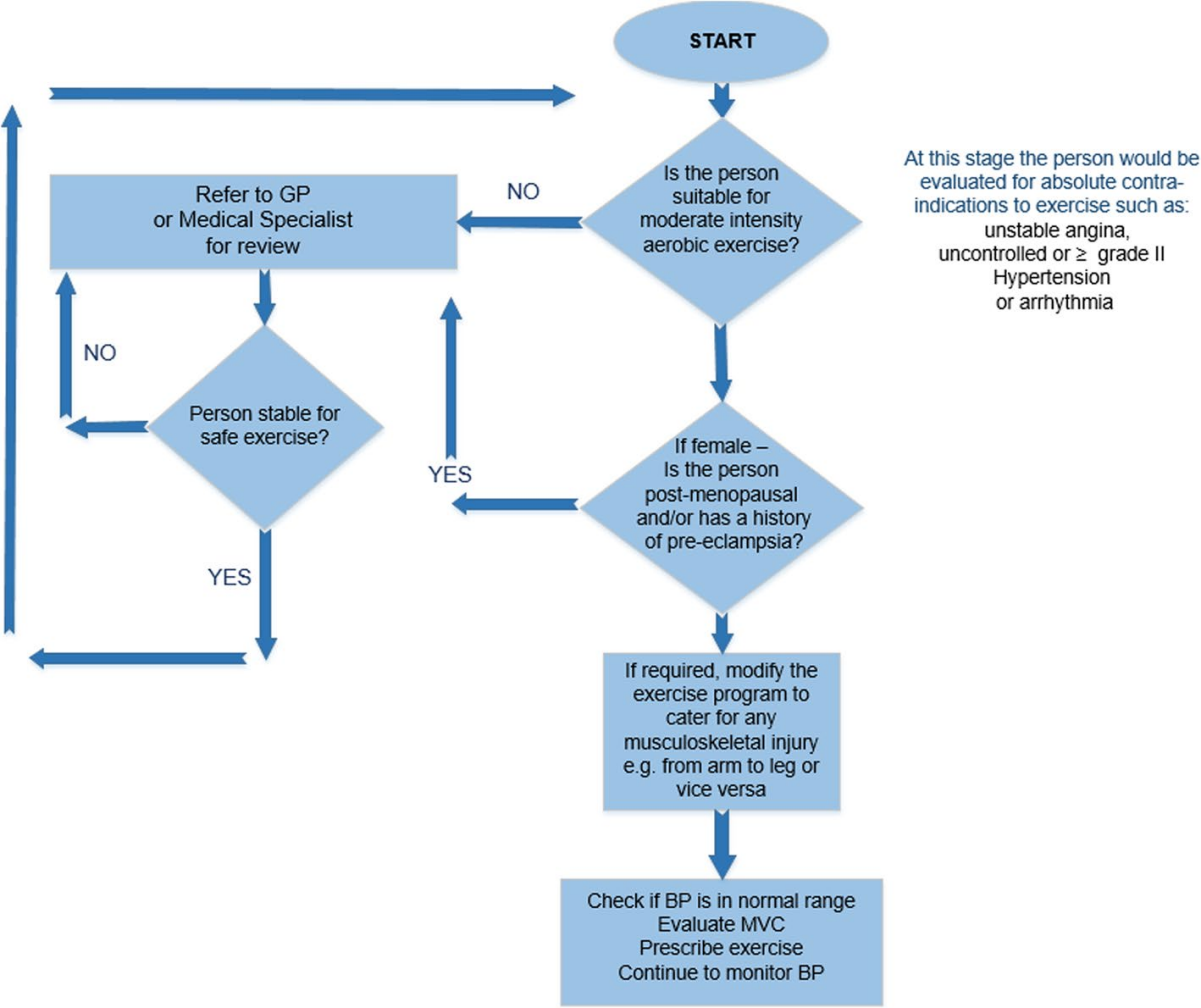
Triage flowchart for assessing a person with hypertension for isometric resistance training exercise programming. GP, general practitioner; BP, blood pressure; MVC, maximal voluntary contraction

As is recommended for the commencement of any new exercise training, individuals should be adequately screened and cleared by a suitably qualified health professional. In prescreening and assessing an individual to ascertain suitability for IRT, certain questions can be asked by an exercise specialist to assist in guiding patients in selecting an appropriate IRT protocol (Fig. 4).

Questions an exercise specialist must ask or consider are the following: (1) Would this person be suitable for moderate intensity aerobic exercise? If yes, then they are probably suitable for IRT from a CV standpoint. Obvious red flags requiring further clinical evaluation include unstable angina, grade II or higher hypertension that is uncontrolled, or arrhythmia, but these flags are relevant to any form of exercise. If no, then it is recommended to wait until the patient is treated/stabilized before proceeding. (2) In women, is the person over 50 years (postmenopausal) and/or has a history of preeclampsia? If so, contact the medical practitioner and ask about other risk factors. (3) Is there any musculoskeletal injury that may require modification of the IRT protocol, from arm to leg or vice versa?

#### Prescription and delivery of IRT

Table 4 illustrates an IRT exercise prescription model for translation to clinical practice for BP management.

**Table 4:** Translation of IRT into clinical practice: model of exercise prescription for BP management

Variable	Recommended for nonclinical and (supervised) clinical populations^a)^	
	Upper limb	Lower limb
Mode	Handgrip; unilateral of bilateral	Wall squat (leg press)
Frequency	3 times/wk	3 times/wk
Intensity	30% MVC^b)^	15%–25% MVC^c)^
Time	4 × 2-min contractions (handgrip squeeze); separated by 3-min rest periods	4 × 2-min contractions (hold); separated by 2-min rest periods
Special consideration	Session should be initially supervised by an exercise professional, and progressed to a HEP when feasible Breathe normally (to avoid Valsalva pressor response) Do not exceed 30% MVC (counterproductive) Individuals unable to sustain the 2 min at 30% MVC should commence with 15%–20% MVC	Session should be initially supervised by an exercise professional, and progressed to a HEP when feasible Breathe normally (to avoid Valsalva pressor response) Monitoring of HR, BP, and training dose Do not exceed 30% MVC (counterproductive) Individuals unable to sustain the 2 min should commence with 10%–15% MVC

*IRT* Isometric resistance training, *BP* Blood pressure, *MVC* Maximal voluntary contraction, *HEP* Home-based exercise program, *HR* Heart rate

^**a)**^As with the commencement of any exercise program, individuals should be adequately screened and cleared by a suitable qualified exercise professional

^b)^The 30% MVC should be established prior to the beginning of each session by completing two to three MVC to establish correct MVC value to be used for the session

^c)^Wall squat exercise are performed at a participant-specific knee joint angle relative to a target HR of 95% peak HR. Isometric wall squat exercise intensity can be reliably adjusted by manipulating knee joint angle [50, 51]

Upper limb IRT can be prescribed simply with the use of a hand dynamometer, numerous models are available, ranging from expensive research grade to simple low technology versions. As long as the dynamometer can accurately record the force generated and display maximum force generated (100% MVC) it can be used to prescribe handgrip IRT, we suggest using 30% MVC and retesting MVC and recalculating 30% MVC, every session.

Based upon current practice, lower limb IRT is more difficult to prescribe using a low technology approach. Researchers have previously demonstrated a reliable linear relationship between lower limb intensity (at that point it was defined by vastus lateralis surface electromyography) and exercising HR measured during an incremental isometric exercise test performed using double-leg extension. HR was shown to be relatively stable in the last 30 s of each 2-min workload, allowing reliable prescription from the final 2-min workload reached [51]. More recently, a similar robust (inverse) relationship was demonstrated between HR and knee joint angle during a laboratory-based incremental wall squat test [64]. This allows for an effective home-based isometric wall squat prescription with accurate control of exercise intensity based upon the knee joint angle required to elicit a given percentage (normally 95%) of HR peak/mean HR during the final 30 s of the incremental test [13, 15].

Since the adoption of lower limb IRT is currently limited by the need for an initial laboratory-based assessment to allow an individualized prescription, we propose that a first bout rating of perceived exertion (RPE) response of five to six out of 10 on the modified Borg scale is probably a simpler and equally reliable method to ascertain the correct lower limb intensity (%MVC). Indeed, recent publications by Lea et al. [83] assessed the validity and reliability of RPE as a measure of intensity during isometric wall squat exercise and have shown that the Isometric Exercise Scale (IES) provides valid and reliable measurements of RPE, exercise intensity; and IES results had strong positive relationships with the criterion measures of the changes in physiological exertion (HR and BP) during continuous incremental isometric exercise interventions [84]. Thus, the IES could prove to be useful in selecting and monitoring of workloads for those (especially vulnerable populations) wishing to utilize isometric wall squat exercise, or other IRT interventions.

### Key clinical issues

Time, accessibility, and cost are commonly reported barriers to exercise participation and adherence [85–87]. The evidence for the benefits of IRT in improving general health including chronic conditions is unequivocal. Given the low rate of participation and the problem of long-term adherence, where possible, exercise specialists need to consider interventions that can reduce barriers, promote adherence and in turn have patients reap the benefits. IRT has the potential to do this. It is accessible to most people, takes as little as 17 min per session three times weekly, and the cost is negligible, especially when appropriately transitioned to a home exercise program.

## Conclusions

The aim of this work was to provide a summary of the available evidence as to the efficacy and safety of IRT and how this can best be transitioned into a real-world clinical setting. A rapidly expanding evidence base indicates IRT to be very effective for managing hypertension. There is no published data suggesting IRT is unsafe at prescribed MVC intensities typically used to elicit antihypertensive effects. Published data in potentially high-risk populations (e.g., heart failure) shows relevant benefits beyond BP lowering (e.g., improved endothelial and collateral vessel function). The size of BP reductions which have consistently been shown equate to > 10% myocardial infarction and > 20% stroke reductions. However, despite the recent American College of Cardiology and American Heart Association endorsement of IRT [29], to date it has not been widely accepted and is underused globally as an adjunct treatment, indicating that translation into practice may require resourcing to improve awareness and assist with delivery.

## Data Availability

Not applicable.

## Abbreviations

AET: Aerobic exercise training
ARR: Absolute risk reduction
bpm: Beats per minute
BP: Blood pressure
CI: Confidence interval
CV: Cardiovascular
DBP: Diastolic blood pressure
DRT: Dynamic resistance training
HEP: Home-based exercise program
HR: Heart rate
IES: Isometric Exercise Scale
IPD: Individual patient data
IRT: Isometric resistance training
MAP: Mean arterial pressure
MVC: Maximal voluntary contraction
N: Newton
NNT: Number needed to treat
RCT: Randomized controlled trial
RPE: Rating of perceived exertion
RPP: Rate pressure product
SBP: Systolic blood pressure
VEGF: Vascular endothelial growth factor
